# Accelerated Electro-Optic Switching in Liquid Crystal Devices via Ion Trapping by Dispersed Helical Carbon Nanotubes

**DOI:** 10.3390/mi16040457

**Published:** 2025-04-12

**Authors:** Rajratan Basu, Christian C. Kehr

**Affiliations:** Soft Matter and Nanomaterials Laboratory, Department of Physics, The United States Naval Academy, Annapolis, MD 21402, USA

**Keywords:** liquid crystals, ionic impurities, helical carbon nanotubes, electro-optic effects

## Abstract

Free ion impurities in liquid crystals significantly impact the dynamic electro-optic performance of liquid crystal displays, leading to slow switching times, short-term flickering, and long-term image sticking. These ionic contaminants originate from various sources, including LC cell fabrication, electrode degradation, and organic alignment layers. This study demonstrates that doping LCs with a small concentration of helical carbon nanotubes effectively reduces free ion concentrations by approximately 70%. The resulting reduction in ionic impurities lowers the rotational viscosity of the LC, facilitating faster electro-optic switching. Additionally, the purified LC exhibits enhanced dielectric anisotropy, further improving its performance in display applications. These findings suggest that helical carbon nanotubes doping offers a promising approach for mitigating ion-related issues in liquid crystals without the need for additional chemical treatments, paving the way for an efficient liquid crystal display technology.

## 1. Introduction

Excess ionic impurities in liquid crystals (LCs) present significant challenges [1,2] in liquid crystal display (LCD) technology, affecting electro-optic performance through slow response times, short-term flickering, and long-term image retention (commonly known as image sticking or dead pixels) [3,4,5,6,7,8,9]. These free ions originate from multiple sources, including LC chemical synthesis, polyimide (PI) alignment layers, and the degradation of indium tin oxide (ITO) conductive electrodes [10,11,12]. Additionally, ionic contamination can arise from the chemical decomposition and self-dissociation of LC materials [13]. Furthermore, LC mixtures containing cyano compounds, such as E7, exhibit a tendency to adsorb more ions due to the cyano polar group. Consequently, for applications in thin-film transistor liquid crystal displays (TFT-LCDs), the predominant LC compounds are fluorinated to mitigate ion absorption [14,15]. Understanding the effects of free ion impurities on the electrical, mechanical, and electro-optic properties of LCs is crucial for both fundamental research and practical applications, as the presence of ions can influence the anchoring energy, elastic constants, and switching responses [16,17,18,19,20,21,22,23]. 

Several methods exist to mitigate ionic contamination in LCs [24]. Traditional purification techniques, such as vacuum sublimation, chromatography, extraction, electrodialysis, multiple recrystallizations, vacuum distillation, zone refining, and ion exchange, are effective but often costly, time consuming, and labor intensive [25,26,27,28]. Furthermore, even highly purified LCs can acquire new ionic impurities during device fabrication due to interactions with electrodes, alignment layers, and adhesives [10,11,12]. Thus, developing alternative approaches to ion removal remains a key area of ongoing research.

Recent efforts have explored nanomaterials as an alternative to conventional chemical purification. Studies have shown that dispersing nanomaterials such as ferroelectric nanoparticles [29,30], titanium nanoparticles [31], carbon nanotubes [32,33], graphene [34,35,36,37,38], fullerenes [36,39,40], and gold nano-urchins [41] in LCs can effectively reduce free ion concentrations via ion trapping. Moreover, two-dimensional materials such as graphene [42] and hexagonal boron nitride [43,44], as alignment agents, have shown ion-capturing abilities that improve the operation of electro-optic LC devices.

Helical carbon nanotubes (*h*CNTs) [45] represent a unique class of chiral materials with a distinctive spring-like morphology, high modulus, and superior electronic properties compared to conventional graphite structures [46,47]. This study demonstrates that doping LCs with a small concentration of *h*CNTs results in a substantial (~70%) reduction of free ions. The trapping mechanism is attributed to the anisotropic, spring-like outer surface of the *h*CNTs, which effectively captures ionic impurities. Experimental results reveal that this ion reduction leads to notable changes in the intrinsic properties of the LC, including decreased rotational viscosity, improved electro-optic switching dynamics, and enhanced dielectric anisotropy. 

From a microscale technology perspective, this approach offers a tunable pathway for improving LC-based displays and photonic devices by controlling ion transport dynamics at the nanoscale. The experimental validation of this mechanism within a well-characterized LC cell architecture advances the understanding and engineering of microstructured LC devices with enhanced electro-optic properties. By presenting an effective strategy for ion management in LC systems, this work paves the way for next-generation high-speed liquid crystal devices, making it relevant to micro and nanoscale technologies.

## 2. Materials

**Figure 1:**
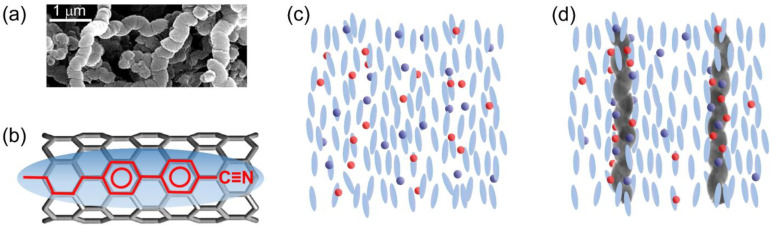
(**a**) An SEM image of *h*CNTs. (**b**) A schematic representation of the LC-CNT interaction: anchoring of a liquid crystal molecule on a carbon nanotube surface due to π–π electron stacking. The blue ellipsoid represents a generic liquid crystal molecule, while the black cylindrical honeycomb structure depicts the carbon nanotube surface. (**c**) Random distribution of free ions in a nematic phase. (**d**) *h*CNTs’ ion trapping process in a nematic phase.

In this study, non-functionalized helical multiwalled carbon nanotubes (*h*CNTs) in powder form, obtained from US Research Nanomaterials, Inc. (Houston, TX, USA), were utilized to investigate their effect on ionic impurity reduction and electro-optic behavior in a nematic LC system. The *h*CNTs had an outer diameter of 130 nm and an average length of 6 μm. Figure 1a is an SEM image of *h*CNTs.

The *h*CNTs were initially dispersed in ethanol to ensure a uniform dispersion of *h*CNTs in the LC medium. The ethanol+*h*CNTs suspension was first shaken using a vortex mixer for 1 h and subsequently sonicated for 6 h. The nematic LC E7 (EMD Millipore Corporation, Billerica, MA, *T*_NI_ = 60 °C) was then added to the ethanol+*h*CNTs mixture and sonicated for an additional hour to achieve complete dissolution. The ethanol was slowly evaporated at an elevated temperature, leaving behind a purified E7 + *h*CNTs mixture with a final *h*CNT concentration of approximately 3.1 × 10⁻^3^ wt.%. To eliminate residual solvent and air bubbles, the mixture was degassed under vacuum for 4 h, followed by further sonication for 6 h.

For consistency, the pure LC E7 was subjected to the same treatment process—dissolution in ethanol, controlled evaporation, and degassing—before experimental analysis. The LC E7 and E7 + *h*CNTs mixtures were then introduced into commercially manufactured antiparallel-rubbed capacitive type planar LC cells (Instec, Inc., Boulder, CO, USA), featuring an ITO-coated area of 1 × 1 cm^2^ and a cell gap of *d* = 20 ± 0.20 μm. An optical interferometric measurement was used to accurately measure the cell thickness using the equation, *d* = *k λ*_1_ *λ*_2_*/*2*(λ*_2_ − *λ*_1_*)*, where *k* represents the number of interference cycles between wavelength*s λ*_1_ and *λ*_2_. The cells were filled via capillary action at an elevated temperature (*T* = 65 °C) in the isotropic phase and subsequently cooled to room temperature for characterization. 

It is well established that LC molecules anchor to the surface of *regular* CNTs via π–π stacking interactions, maximizing the hexagon–hexagon interaction between the LC molecules’ benzene rings and the honeycomb lattice of the CNTs [48]. This interaction is schematically illustrated in Figure 1b, where the ellipsoid depicts a generic LC molecule, and the black honeycomb cylindrical structure denotes the CNT surface. The molecular structure of a representative LC molecule is depicted within the ellipsoid, positioned on the CNT surface to highlight the π–π electron stacking interaction. The alignment of the benzene rings of the LC molecules with the hexagonal lattice of the CNTs characterizes this interaction. The anchoring energy associated with this π–π stacking interaction is estimated to be |*U*_anchor_| ≈ 2.0 eV per molecule [48]. When CNTs are dispersed as colloidal inclusions in an LC medium, this π–π stacking interaction drives the CNTs to align along the nematic director while simultaneously inducing the LC molecules to orient along the CNT long axis on the CNT surface [49,50,51,52]. This stable anchoring mechanism leads to several intriguing phenomena, including an enhancement of the nematic orientational order parameter in LCs [51], the formation of pseudo-nematic LC domains around the CNTs even in the isotropic phase [52], the transfer of CNT surface chirality to otherwise achiral LCs [53,54,55], an increase in the polar anchoring energy at the LC–CNT interface [56], and vertically aligned CNTs-induced homeotropic LC alignment [57,58]. 

All those prior studies have focused on regular (non-helical) CNTs. The findings presented in this work demonstrate the potential of *h*CNTs as a promising approach for mitigating ion-related issues in LCs without necessitating extensive chemical purification. Figure 1c illustrates ions in a nematic phase. Figure 1d depicts ion trapping by dispersed *h*CNTs in the LC.

## 3. Experiments, Results, and Discussion

### 3.1. Ion Concentration

The free ion concentration, n_i_, in LC E7 and E7 + *h*CNTs, was determined by analyzing the transient ion current, *I*_ion_, generated when the applied voltage polarity across the cell is inverted [30,59]. When a square-wave voltage alternates between +*V* and −*V*, the LC molecules do not rotate since their reorientation depends on the electric field magnitude, not its polarity [49,50,51,52,60]. However, voltage polarity inversion induces free ions to migrate toward opposite electrodes, resulting in a transient ion current within the LC cell.

**Figure 2:**
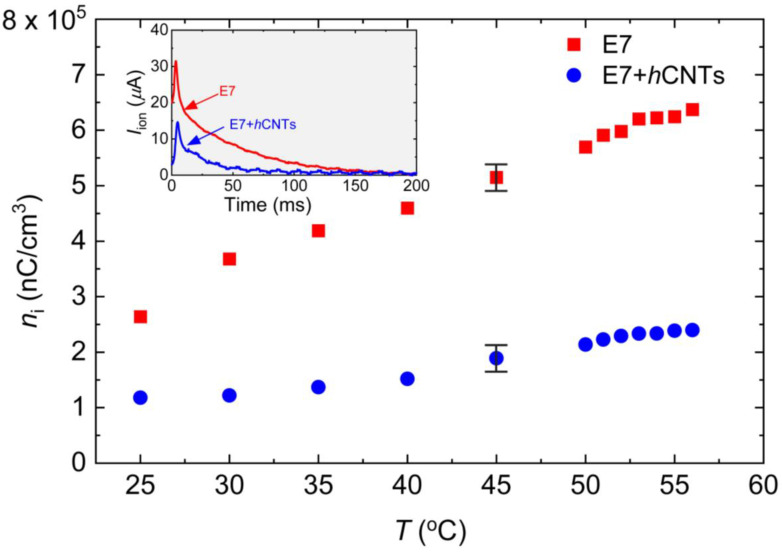
Free ion concentration, *n*_i_, as a function of temperature for E7 and E7 + *h*CNTs samples listed in the legend. Typical error bars are shown. Inset: ion current, *I*_ion_ as a function of time for E7, and E7 + *h*CNTs at 25 °C after the voltage is inverted across the cells. The peak represents the ion bump when positive and negative ions meet in the middle of the cell.

To generate *I*_ion_, a square-wave voltage with a peak-to-peak amplitude of 30 V (ranging from +15 V to −15 V) at 1 Hz was applied using an automatic liquid crystal tester (Instec, Inc., Boulder, Colorado, USA). The resulting ion current as a function of time was recorded at T = 25 °C, as shown in the inset in Figure 2. The transient ion current reaches its peak value when the positive and negative ions meet approximately at the center of the cell. The peak time is given by the following: $t_{ion-peak}=\frac{d^{2}}{2\mu E}$, where *μ* is the ion mobility [59]. As the ions continue their migration, *I*_ion_ eventually decays to zero when they reach the opposite electrodes, as observed in the inset in Figure 2. The total free ion transport was calculated by integrating the area under the *I*_ion_ vs. time curve, and the free ion concentration was extracted using the following: $n_{i}=(\int_{0}^{t}I_{ion}dt)/A.d$, where A is the active electrode area, and *d* is the cell gap. At elevated temperatures, both the free ion concentration and ion mobility increase. Figure 2 shows *n*_i_ for both samples as a function of temperature, revealing a substantial suppression of ion concentration in E7 + *h*CNTs compared to pure E7. Notably, for the E7 + *h*CNTs sample, *n*_i_ is reduced by approximately 60% to 70% in the temperature range, 25 °C to 60 °C.

Literature reports [61] indicate that non-helical CNTs at a concentration of 0.05 wt.% reduce the ion concentration in liquid crystal E7 by approximately 50% at 30 °C. However, at temperatures exceeding 45 °C, this reduction decreases to around 20% or less [61]. In the present study, *h*CNTs at an ultralow concentration of ~10^−3^ wt.% achieved a reduction in ion concentration exceeding 65% over a broad temperature range. Since no known attractive force exists between *h*CNTs and free ions, this enhanced ion trapping is likely facilitated by the asymmetric spring-like morphology of *h*CNT-walls, which effectively capture both positive and negative ions within the LC.

The ion-trapping efficiency of *h*CNTs can be better understood by comparing it with other nanomaterials reported in the literature. Previous studies have demonstrated that different nanoparticles exhibit varying degrees of ion-trapping capabilities in liquid crystals. For instance, graphene nanoplatelets at a concentration of 0.5 wt.% in LC 8OCB resulted in a 30% reduction in ion concentration [24], while a similar concentration of graphene platelets in cholesteric LCs led to an approximately 32% reduction [24]. A much higher efficiency was observed in ferroelectric LCs doped with 0.5 wt.% fullerenes (C_60_), where an 80% decrease in ion concentration was reported [40]. Ferroelectric BaTiO_3_ nanoparticles, when incorporated at 0.275 wt.% in LC 5CB, were found to trap about 50% of the free ions [30]. In another study [24], titanium dioxide (TiO_2_) nanoparticles at a concentration of 0.1 wt% were observed to reduce ion impurities in LC E7 by 53%. 

Thus, the ion trapping by *h*CNTs in the current study is comparable to, or in some cases even exceeds, that of other nanomaterials reported in prior studies. However, what sets *h*CNTs apart is their ability to achieve a significant reduction in ion concentration at substantially lower doping concentrations than other nanoparticles. 

The *h*CNTs utilized in this study had an average length of 6 μm. The cell gap employed is 20 μm, which is larger than the length of the *h*CNTs. However, to achieve a rapid response time, the LC cell gap is typically 5 μm in most LCD applications. Consequently, shorter *h*CNTs are required for thinner cells. It has been demonstrated that the electrochemical shortening process can effectively truncate CNT lengths to as small as 500 nm [62]. This process is also applicable to *h*CNTs since it is not dependent on any specific type of CNT. Therefore, electrochemically shortened *h*CNTs can be used for thin LC cells.

### 3.2. Rotational Viscosity

**Figure 3:**
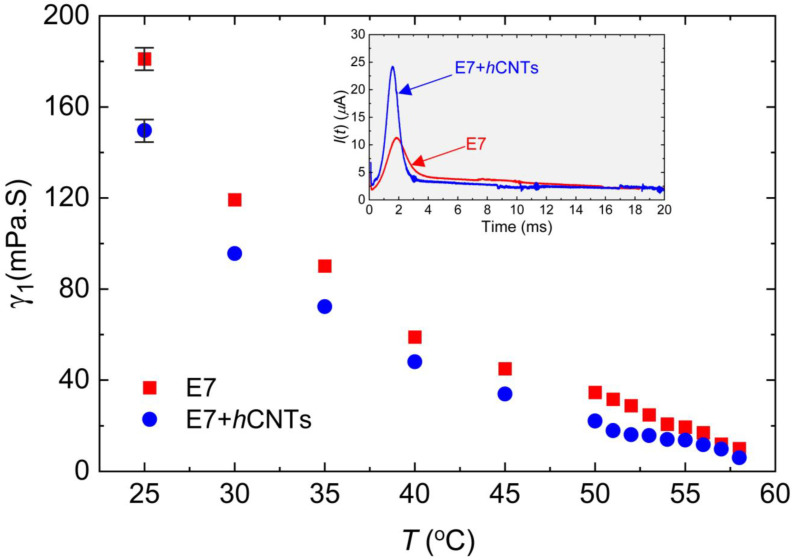
Rotational viscosity, *γ*_1_ as a function of temperature for E7 and E7 + *h*CNTs samples, listed in the legend. Typical error bars are shown. Inset: transient current, *I*(*t*) as a function of time for E7 and E7 + *h*CNTs at *T* = 25 °C.

The rotational viscosity, *γ*_1_, of an aligned LC quantifies the internal friction among LC molecules during their rotational motion. To investigate *γ*_1_ in our LC samples, we conducted experiments using the same planar-aligned capacitive cell configuration, where the transient current induced by a DC field was measured [63,64,65]. When a DC field, significantly exceeding the threshold field, is applied across the LC cell, the induced current *I*(*t*) exhibits a characteristic time response as the nematic director undergoes dynamic rotation. The time-dependent current response follows the relation

(1) $$I\left(t\right)=\frac{A{\left(\Delta \varepsilon \varepsilon_{o}\right)}^{2}E^{3}}{\gamma_{1}}{sin}^{2}[2\theta \left(t\right)]$$

where *A* is the area of the cell, *E* is the applied electric field, Δ*ε* is the dielectric anisotropy, *ε*_o_ is the free space permittivity, and *θ* represents the director angle relative to the electrodes at a given time. The peak current occurs at *θ* = 45°, yielding

(2) $$I_{p}=\frac{A{\left(\Delta \varepsilon \varepsilon_{o}\right)}^{2}E^{3}}{\gamma_{1}}$$

at the peak time,

(3) $$t_{p}=[\frac{\gamma_{1}(-ln(\tan\theta_{o}))}{\Delta \varepsilon \varepsilon_{o}}]\frac{1}{E^{2}}$$

where *θ*_o_ is the pre-tilt angle. A DC voltage pulse of 35 V with a 1 Hz interval was applied across the cell to generate *I*(*t*), which was subsequently detected as a function of time via a digital storage oscilloscope through a load resistor in series. The inset in Figure 3 presents an example of *I*(*t*) for two test cells, E7 and E7 + *h*CNTs, at *T* = 25 °C. The peak current *I*_p_ was extracted from the *I*(*t*) versus time graph, and *γ*_1_ was determined using the known values of *E*, Δ*ε*, and *A*. The method for measuring Δ*ε* is discussed later.

Figure 3 depicts *γ*_1_ as a function of temperature for the two cells. A clear pre-transitional behavior is observed for both samples, with E7 + *h*CNTs exhibiting a significant reduction in *γ*_1_ compared to pure E7. The reduction in *γ*_1_ for the E7 + *h*CNTs sample is approximately 17% at *T* = 25 °C.

Previous studies have suggested that the suppression of ionic impurities can lead to a decrease in the rotational viscosity of an LC. For instance, quantum dot-doped LCs have been shown to exhibit reduced *γ*_1_ due to the trapping of ionic impurities, which lowers the overall ionic density and resistance of the nematic medium [66]. Similarly, our group previously reported that graphene flakes in a ferroelectric LC reduce rotational viscosity by effectively trapping ionic impurities [34]. Another study demonstrated that titanium nanoparticles suppress free ions, leading to stronger van der Waals dispersion interactions between LC molecules and the alignment layers, thereby reducing the pre-tilt angle of the LC molecules [31]. According to Equation (3), $\gamma_{1}\propto 1/(-ln(\tan\theta_{o}))$, for a constant applied field, implying that a decrease in the pre-tilt angle, *θ*_o_, results in a corresponding decrease in *γ*_1_. In our study, we attribute the observed reduction in *γ*_1_ for the E7 + *h*CNTs sample to the suppression of ionic impurities. The decrease in internal resistance and friction, coupled with enhanced van der Waals interactions between LC molecules and the alignment layers, likely leads to a reduction in the pre-tilt angle, thereby decreasing *γ*_1_. While a direct quantitative theoretical model linking ion concentration to *γ*_1_ is not yet available, our findings are in agreement with previous reports, suggesting a coherent relationship between reduced ionic impurities and lower rotational viscosity.

### 3.3. Dielectric Anisotropy

**Figure 4:**
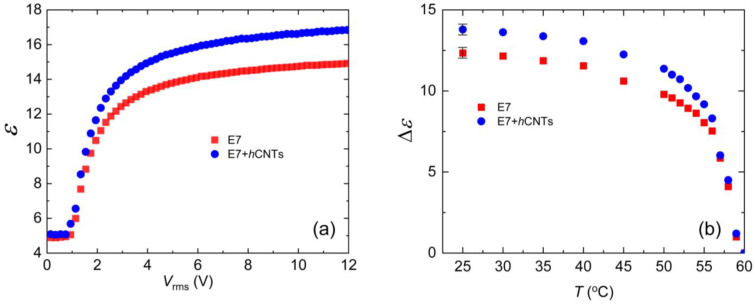
(**a**) Dielectric constant, *ε* as a function of *V*_rms_ for E7 and E7 + *h*CNTs samples, listed in the legend at *T* = 25 °C. (**b**) Dielectric anisotropy, Δ*ε* as a function of temperature for E7 and E7 + *h*CNTs samples, listed in the legend. Typical error bars are shown.

The nematic phase exhibits dielectric anisotropy, defined as Δ*ε* = *ε*_∥_−*ε*_⊥_, *ε*_∥_ and *ε*_⊥_, and are the dielectric permittivities parallel and perpendicular to the nematic director, respectively. To measure the dielectric constant *ε* as a function of the electric field, we employed an automatic liquid crystal tester (Instec, Inc., Boulder, CO, USA) operating at 1000 Hz for both pure E7 and E7 + *h*CNT samples. Figure 4a shows *ε* vs. V_rms_ at 25 °C. The values of *ε*_∥_ and *ε*_⊥_ were then used to determine Δ*ε*, as described in detail elsewhere [67]. Figure 4b illustrates the temperature dependence of Δ*ε* for the two test cells. Notably, Δ*ε* is higher in E7 + *h*CNTs sample.

According to Maier and Meier’s theory [68], the dielectric anisotropy Δ*ε* in the nematic phase is given by

(4) $$∆\varepsilon =\frac{NhFS}{\varepsilon_{0}}\left[∆\alpha +\frac{\mu^{2}F}{2k_{B}T}\left(3\cos^{2}\beta -1\right)\right]$$

where *N* is the number density of LC molecules, *μ* is the resultant dipole moment, Δ*α* is the polarizability anisotropy, *S* is the orientational order parameter, *β* is the angle between the molecular long axis and the dipole moment, *h* is the cavity field factor, and *F* is the feedback factor [68].

The presence of excess ionic impurities in the LC can influence the effective dipole moment of the LC molecules. Since LC molecules possess permanent dipole moments, negatively charged ions may accumulate near the positive ends of the molecules, while positively charged ions may cluster near the negative ends. This ionic accumulation effectively reduces the net dipole moment μ, leading to a decrease in Δ*ε* according to Equation (4). Thus, we propose that the suppression of ionic impurities in the E7 + *h*CNTs sample enhances the effective polarity of the LC molecules, resulting in an increased Δ*ε*. 

Previous studies support this interpretation. For example, it has been reported that doping a nematic LC with 0.2 wt.% TiO_2_ nanoparticles increases Δ*ε*, an effect attributed to the ion-trapping capability of TiO_2_ [69]. Another study [66] demonstrated that doping nematic LCs with quantum dots (0.05 wt.%) captures free ions, enhancing the LC’s birefringence. This suggests that suppressing ionic impurities improves the orientational order parameter S, which, in turn, leads to an increase in Δ*ε*. Our observations are consistent with these findings, further supporting the role of ion trapping in enhancing the dielectric anisotropy of liquid crystal systems. 

### 3.4. Electro-Optic Effect

**Figure 5:**
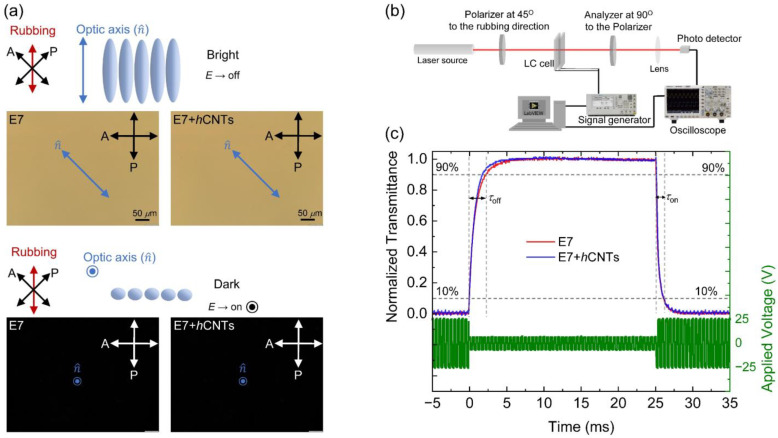
(**a**) A schematic representation of the field-off bright state and field-on dark state for a nematic LC. The micrographs for E7 and E7 + *h*CNTs cells show the field-off bright state and the field-on dark state, respectively. (**b**) A schematic representation of the electro-optic experimental setup. (**c**) Dynamic electro-optic response in E7 and E7 + *h*CNTs filled test cells. The driving modulated square wave voltage profile at *f* = 20 Hz is indicated on the right-hand *y*-axis. The left-hand *y*-axis shows the normalized transmitted intensity over time as *V* is turned off (at *t* = 0) and then turned on (at *t* = 25 ms), for the two test cells, as identified in the legend at *T* = 25 °C.

The rotational viscosity, *γ*_1_, plays a critical role in determining the dynamic electro-optic response of aligned nematic LCs. Since *γ*_1_ is altered in the presence of *h*CNTs, we investigated the dynamic electro-optic response of the E7 + *h*CNTs composite and compared it to that of pure E7. First, Figure 5a shows the field-off bright state and field-on dark state with their corresponding micrographs for the E7 and E7 + *h*CNTs composites. No significant changes were observed in the micrographs as there was no visible aggregation for the E7 + *h*CNTs composite. Then, an experimental approach, schematically shown in Figure 5b was employed using an optical setup where a 5-mW He-Ne laser beam (λ = 633 nm) was directed through a polarizer, an LC cell (with the director oriented at 45^o^ relative to the polarizer), a crossed analyzer, and a photodetector. The detected intensity was recorded using a digital storage oscilloscope to analyze the temporal variation in transmittance upon applying a modulated square-wave driving voltage at 25 °C. Upon voltage application, the transmitted intensity decreases, with the optical switching on time (*τ*_on_) defined as the interval required for intensity to drop from 90% to 10% of its maximum value. Conversely, upon voltage removal, the transmitted intensity increases, with the optical switching off time (*τ*_off_) defined as the time required for intensity to rise from 10% to 90% of its maximum value. These switching times are governed by the following relations [70]:

(5) $$\tau_{\mathrm{on}}\propto \frac{\gamma_{1}}{\Delta \varepsilon \varepsilon_{o}V^{2}-K_{11}\pi^{2}}(d^{2}+\frac{4dK_{11}}{W_{\theta }});\tau_{\mathrm{off}}\propto \frac{\gamma_{1}}{{\Delta \varepsilon \varepsilon_{o}V_{b}^{2}-K}_{11}\pi^{2}}(d^{2}+\frac{4dK_{11}}{W_{\theta }})$$

where *d* represents the cell gap, Δ*ε* is the dielectric anisotropy, *V* (>>*V*_th_) is the driving applied voltage, *V*_b_ is the bias voltage, *ε*_0_ is the permittivity of free space, *K*_11_ is the splay elastic constant, and *W*_θ_ is the polar anchoring strength coefficient. 

Figure 5c illustrates the normalized transmitted intensity (left-hand *y*-axis) for E7 and E7 + *h*CNTs as a function of time upon applying a modulated square-wave driving voltage (right-hand *y*-axis) with a |*V*_b_| = 5 V and a |*V*|= 25 V (>>*V*_th_ = 0.90 V) and *f* = 20 Hz. 

Table 1 reveals a small decrease (~4%) in *τ*_on_ for the E7 + *h*CNT sample, whereas *τ*_off_ exhibits a significant acceleration (~25%) for the E7 + *h*CNT sample, as corroborated by Figure 5c. This enhancement in switching response is primarily attributed to the substantial reduction in *γ*_1_ in the E7 + *h*CNT system. In this study, *V*_th_ ≈ 0.90 V and the driving voltage, |*V*| = 25 V, ensuring *V* >> *V*_th_. Under such high-voltage conditions, the electro-optic switching operates in the transient nematic relaxation mode, where *τ*_off_ is inherently fast (~milliseconds), even for large cell gaps [71,72]. Furthermore, since *V* >> *V*_th_, the driving voltage predominantly dictates *τ*_on_, as indicated by Equation (5), resulting in a minimal change in *τ*_on_ for the E7 + *h*CNT sample compared to pure E7. However, upon deactivation of the driving voltage, *τ*_off_ is a diffusion-type relaxation, mainly governed by the elastic interactions between the LC and the planar alignment layers and influenced by *γ*_1_. Consequently, the observed reduction in *γ*_1_ leads to a significantly faster *τ*_off_ in the E7 + *h*CNT sample. Additionally, previous studies [56] indicate that incorporating regular CNTs into an LC can enhance *W*_θ_. Similarly, we propose that *h*CNTs can also enhance W_θ_, leading to a faster *τ*_off_ according to Equation (5).

Figure 5a presents micrographs of E7 and E7 + *h*CNTs cells under a cross-polarized microscope, capturing their voltage-off and voltage-on states. The E7 + *h*CNTs cell’s micrograph at the voltage-off state shows a uniform texture like the E7 cell, with no visible *h*CNT aggregates. This confirms a homogeneous dispersion of *h*CNTs in the LC at the visible length scale.

### 3.5. The Effect of a Higher hCNTs Concentration

As discussed earlier, all presented experiments here thus far have been conducted at a *h*CNT concentration of 3.1 × 10⁻^3^ wt.% in E7. A key question that arises is how the ion-trapping phenomenon is affected by an increase in *h*CNT concentration. To investigate this, we prepared an additional sample with an *h*CNT concentration of 6.4 × 10⁻^3^ wt.% in E7, referred to as E7 + *h*CNTs-2. Experimental results indicate that the ion-trapping efficiency at this higher concentration remains largely unchanged, with only a ±4% variation observed over the studied temperature range.

However, a distinct and intriguing phenomenon was observed at this elevated concentration. It is well established that when conventional CNTs are dispersed as colloidal inclusions in an LC medium, their long axes align along the nematic director [49,50,51,52]. We propose that this alignment mechanism also holds for *h*CNTs. Upon the application of an external electric field, the nematic director reorients along the field direction, and due to their embedding within the nematic matrix, the *h*CNTs follow this field-induced director rotation.

**Figure 6:**
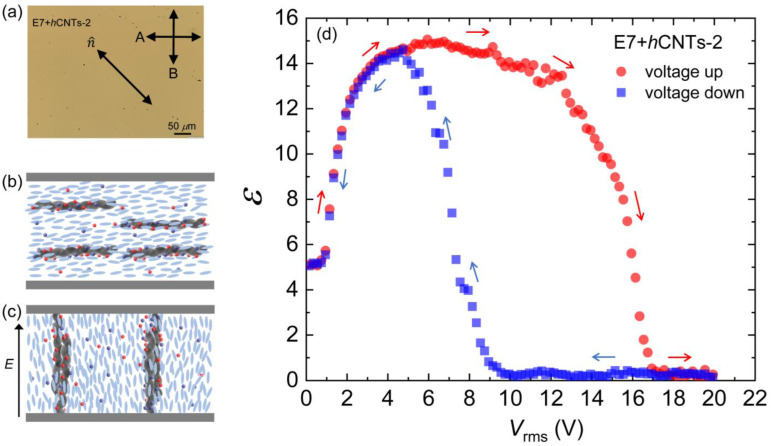
(**a**) A micrograph for the E7 + *h*CNTs-2 cell under a cross-polarized microscope. Small black dots are *h*CNT aggregates. Schematic representation of the presence of *h*CNTs in the LC when (**b**) the field is off and (**c**) the construction of *h*CNT wires bridging the two electrodes of the cell at a high field. (**d**) Dielectric constant, *ε* as a function of *V*_rms_ [voltage cycle up (red arrows) and down (blue arrows)] for E7 + *h*CNTs-2 at *T* = 25 °C.

As the *h*CNTs begin to rotate under the applied field, they exhibit a natural tendency to form wire-like aggregates due to entanglement. At sufficiently high concentrations, such as in E7 + *h*CNTs-2, these *h*CNT-wires become significantly extended. Eventually, under a sufficiently high electric field, the elongated *h*CNT-wires bridge the two electrodes of the LC cell (separated by 20 μm), leading to an insulator-to-conductor transition. Figure 6a shows a micrograph of the E7 + *h*CNTs-2 cell under a cross-polarized microscope, where several small black dots represent *h*CNT aggregates. Figure 6b,c schematically depicts the presence of *h*CNTs in the LC when the field is off, as well as the formation of *h*CNT wires that bridge the two electrodes of the cell under a high field. Figure 6d presents *ε* as a function of *V*_rms_ for E7 + *h*CNTs-2. After reaching a maximum value, *ε* decreases to zero, indicating that the LC cell capacitor has been shorted.

This insulator-to-conductor transition of the LC cell is a reversible process. When the applied field is reduced to zero, the LC cell regains its original capacitance, as illustrated in the voltage up/down cycle shown in Figure 6d. This hysteresis behavior indicates that bridging the two electrodes by the *h*CNT wires occurs at different voltages during the voltage increase and decrease cycles. These findings suggest that 3.1 × 10⁻^3^ wt.% represents an optimal *h*CNT concentration for stable LC operation. Any concentration approaching or exceeding 6.4 × 10⁻^3^ wt.% leads to excessive *h*CNT aggregation, ultimately shorting the cell and preventing normal device functionality. It is important to observe that Figure 4a does not exhibit any hysteresis behavior for either pure E7 or E7 + *h*CNTs. 

## 4. Conclusions

In this study, we have experimentally demonstrated that the incorporation of a small concentration of *h*CNTs into an LC medium effectively reduces the free ion concentration via an ion-trapping mechanism. The resulting decrease in mobile ion density lowers the internal friction within the nematic phase, thereby facilitating faster reorientation of the nematic director under an applied electric field. However, when the *h*CNT concentration exceeds a critical threshold (optimal concentration), the intrinsic entanglement tendency of *h*CNTs leads to the formation of wire-like aggregates, which ultimately short the LC cell.

These findings are significant for mitigating excess ionic impurities in LC materials and highlight the existence of an optimal *h*CNT concentration that balances ion trapping and electro-optic stability. The observed enhancement in optical switching speed is primarily attributed to the reduction in free ion density. This work provides valuable insights into the development of high-performance electro-optic devices based on nanostructured LC composites, offering a potential pathway towards faster and more efficient display and photonic applications.

## Figures and Tables

**Table 1:** The two characteristic times for the pure E7 and E7 + *h*CNTs from Figure 5c.

Samples	*τ*_on_ (μs)	*τ*_off_ (ms)
E7	970	2.40
E7 + *h*CNTs	930	1.80

## Data Availability

The data that support the findings of this study are available from the corresponding author, R.B., upon reasonable request.
